# Effects of Oligomeric Procyanidins From Lotus Seedpod on the Retrogradation Properties of Rice Starch

**DOI:** 10.3389/fnut.2021.751627

**Published:** 2021-09-22

**Authors:** Nianjie Feng, Shaowen She, Hengfeng Hu, Shimiao Tang, Jiangying Tan, Qian Wu, Juan Xiao

**Affiliations:** ^1^Key Laboratory of Fermentation Engineering, Ministry of Education, Hubei Key Laboratory of Industrial Microbiology, National “111” Center for Cellular Regulation and Molecular Pharmaceutics, Hubei Research Center of Food Fermentation Engineering and Technology, Hubei University of Technology, Wuhan, China; ^2^J.S Corrugating Machinery Co. Ltd, Wuhan, China; ^3^State Key Laboratory of Marine Resource Utilization in South China Sea/Ministry of Education, Key Laboratory of Food Nutrition and Functional Food of Hainan Province/Engineering Research Center of Utilization of Tropical Polysaccharide Resources/School of Food Science and Engineering, Hainan University, Haikou, China

**Keywords:** oligomeric procyanidins, retrogradation, rice starch, molecule simulation, hydrogen bonds

## Abstract

The extent of retrogradation strongly affects certain physical and cooking properties of rice starch (RS), which are important to consumers. In this study, oligomeric procyanidins from lotus seedpod (LSOPC) was prepared and used to investigate its inhibitory effect on RS retrogradation. Various structural changes of RS during retrogradation were characterized by differential scanning calorimetry, low field nuclear magnetic resonance, X-ray diffraction, scanning electron microscopy, and Fourier transform infrared spectroscopy. The results showed LSOPC could effectively retard both short- and long-term retrogradation of RS, and its inhibitory effect was dependent on the administered concentration of LSOPC. Molecule simulation revealed the interactions of RS and LSOPC, which indicated that the competition of hydrogen bonds between RS and LSOPC was the critical factor for anti-retrogradation. This inhibitory effect and mechanism of action of LSOPC could promote its applications in the field of starch anti-retrogradation.

## Introduction

Rice is a common food and a basic staple for hundreds of millions of people (1). Starch is the main component of rice, comprising up to about 90% of the dry weight. Retrogradation can substantially affect the physical and cooking properties of starch in the storage (2, 3). It is well known that starch retrogradation can be categorized as short- and long-term. Short-term retrogradation normally occurs in the first few hours after gelatinization and is induced by the reordering and recrystallization of amylose fractions, whereas long-term retrogradation is attributed to the recrystallization of amylopectin fractions that takes place over several days or even weeks.

Several methods have been found to retard or decrease starch retrogradation, such as various modifications and specially designed additives. Extrusion and microwaving are easy-to-operate physical ways, however, the effects of anti-retrogradation are limited (4–6). Acetylation and cross-linking are used as chemical modifications, and provide strong anti-retrogradation effects (7–9). But they are unacceptable for food safety due to the presence of residual chemicals. As for additives, it has been widely shown that retrogradation can be inhibited to a certain degree by adding amylase, saccharides, or emulsifiers (10–12).

In addition, increased awareness of food safety has led to the development of natural food additives. Natural polyphenols have attracted attention because their non-negligible inhibiting effect on starch retrogradation. Rutin, ferulic acid, epigallocatechin gallate and tannin have already been used in research on starch retrogradation (13–16). Procyanidins (PC) are natural polyphenols widely existing in plants, which are formed by the condensation of flavanol monomers (17, 18). The free radical scavenging ability and antioxidant efficiency of PC have generated an enormous interest in the field of natural food additives. As such, PC may offer a new opportunity to inhibit starch retrogradation. However, the interactions between PC and starches remains unclear, and the thorough interactions investigation could provide valuable information for anti-retrogradation effects of PC.

**Scheme 1:**
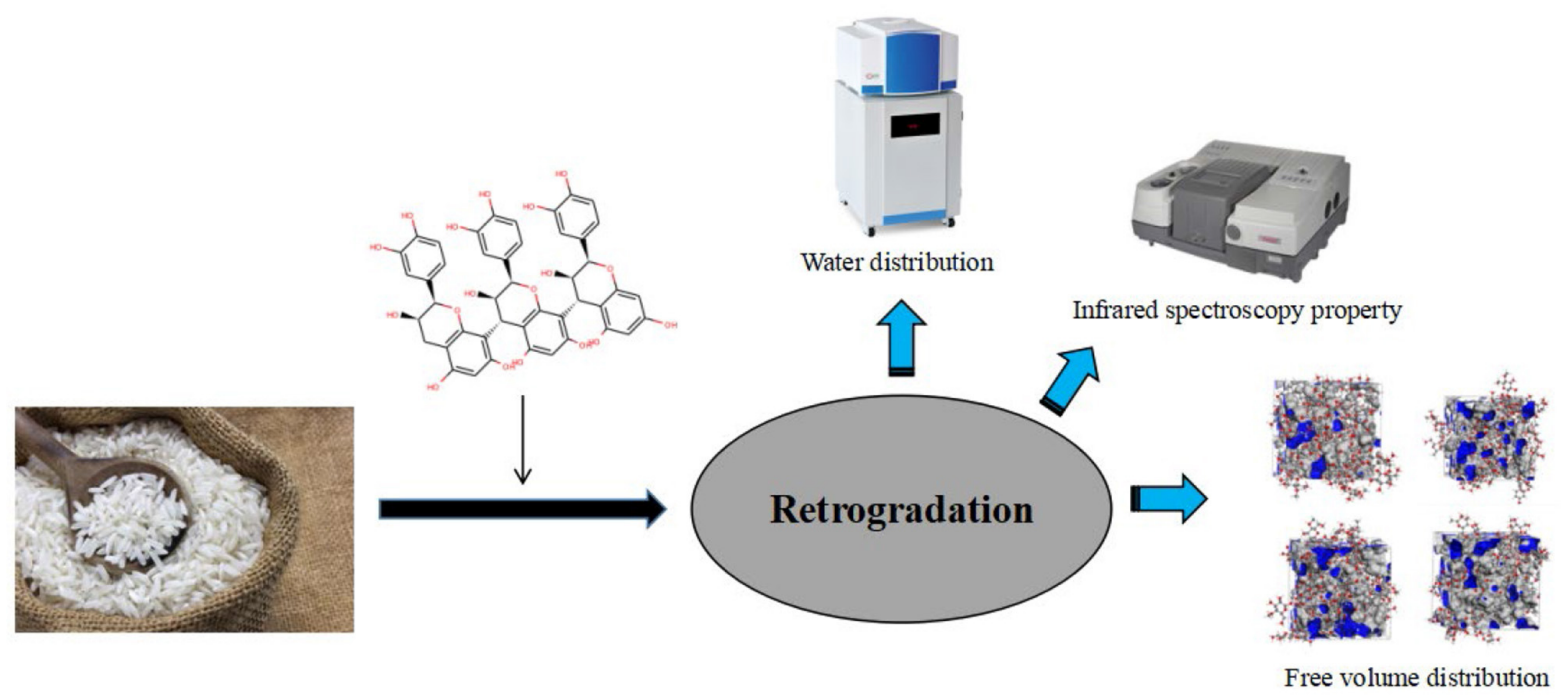
Inhibition illustration of LSOPC on RS retrogradation.

In this study, PC was prepared from lotus seedpod. The retrogradation of rice starch with and without PC was then investigated. Various structural changes of rice starch during retrogradation were characterized by differential scanning calorimetry (DSC), low field nuclear magnetic resonance (LF-NMR), X-ray diffraction (XRD), scanning electron microscopy (SEM), Fourier transform infrared spectroscopy (FT-IR), and computer molecular simulation (Scheme 1).

## Materials and Methods

### Materials

Rice starch (RS) was purchased from Wuxi Jinnong Biotechnology Co., Ltd. (Jiangsu, China). Lotus seedpods were bought from the local supermarket (Wu Zhi 2 hao).

### Preparation of Samples

The method of Wu et al. (19) was used to prepare oligomeric procyanidins from lotus seedpod (LSOPC). The polymerization degree of LSOPC was 3. RS was mixed with LSOPC at the ratios of 100:0, 100:3, 100:6 and 100:9 (RS/LSOPC, w/w). The mixtures were added to deionized water to ensure uniformity, and the mass concentration of RS was 60 g/L.

### Measurement of Thermodynamic Property

A DSC 214 (NETZSCH, Germany) was used to measure the thermodynamic properties of RS with or without LSOPC. The sample (2 mg) was used, and the heating rate is programmed at 10°C/min from 20 to 100°C. After that, the onset temperature (T_onset_), peak temperature (T_peak_) and end temperature (T_end_) were obtained from the pasting curve. The pasting enthalpy (ΔH_g_) was analyzed by the calculation of peak area.

### Detection of Texture Characteristic

After gelatinization, all samples were stored at 4°C for 7 days. The texture characteristics were detected by a TA-XY2i texture analyzer (Stable Micro System Co., United States). The test conditions were as follows: the probe was P/0.5 type, the pre-test, test and post-test speed were all 1.0 mm/s, the trigger force was set to 5.0 g, and the shape variable was set to 40%. TPA software was used to analyze the final results.

### LF-NMR Analysis

Gelatinized samples were stored at 4°C for 7 days. All samples were transferred to the NMR tubes and a NMI 20X NMR imaging analyzer (Shanghai Niumag Co., China) was used to determine the water distribution. The treatment conditions were as follows: the number of sample point was set to 1,024, the number of repeated scans was set to 8, and the relaxation attenuation time was set to 2,000 ms. CPMG pulse sequence was applied to measure the relaxation time.

### FT-IR Analysis

The samples were analyzed by a Nexus 470 FT-IR spectrometer (Nicolet, United States) according to the previous method (20) with some modifications. The samples were freeze-dried, fully ground, and mixed with dried KBr powder. The wavenumber range was 4,000–400 cm^−1^ and the final results were analyzed by omnic 8.0 software.

### XRD Analysis

The crystal structure of the freeze-dried powder sample (10 mg) was measured by a D8-Advance diffractometer (Bruker, United States). The scanning range (2 θ) was 5°-40° and the scanning speed was 2°/min. MDI jade 6.0 software was used to analyze the final results.

### SEM Analysis

About 2 mg of freeze-dried sample was fixed and sprayed gold. Then the sample was observed by a SU-8010 scanning electron microscope (Hitachi, Japan).

### Determination of Dynamic Rheological Property

All samples were cooled to room temperature after gelatinization. A DHR-3 rotational rheometer (TA Instruments Inc., United States) was applied to determine the dynamic rheological properties of samples. The test conditions were as follows: the plate diameter was 40 mm, the gap was 0.5 mm, the temperature was set to 25°C, the scanning strain was set to 1%, and the frequency was 0.1–10 Hz. Finally, the spectra of storage modulus (G') and loss modulus (G”) were obtained.

### Molecule Simulation

The structural models of RS and procyanidins were downloaded from the National Center for Biotechnology Information (NCBI) using PubChem SID: 135332954 and 374367001. ChemOffice 2010 was used to optimize these molecular structures. After that, Materials Studio 8.0 was used to simulate the interactions between RS and procyanidins.

### Statistical Analysis

All data were presented as means ± standard deviation (means ± S.D.). Analyses of variance (ANOVA) were performed by SPSS 25.0. The graph was drawn by OriginPro 8.0.

## Results and Discussion

### Effect of LSOPC on the Thermodynamic Property of RS

The thermodynamic properties are shown in Table 1. Compared with RS without LSOPC, the T_onset_, T_peak_, T_end_ and ΔH_g_ of RS with LSOPC trended downward, and the decline correlated positively to the amount of LSOPC added. This result indicated that RS with LSOPC could gelatinize at a lower temperature, which may be due to the hydroxyl group in procyanidins. The hydroxyl group, which has strong hydrophilicity, could have interacted with the side chain of the starch and then combined with its non-crystalline region. Therefore, the degree of crystallinity changed, and the energy required for starch gelatinization was reduced (21).

**Table 1:** Thermodynamic properties of RS with or without LSOPC.

**Sample**	**T_**onset**_ (^**°**^C)**	**T_**peak**_ (^**°**^C)**	**T_**end**_ (^**°**^C)**	**ΔH_**g**_ (J/g)**
RS	54.37 ± 0.13^a^	79.12 ± 0.24^a^	103.54 ± 0.33^a^	21.46 ± 0.36^a^
RS + 3% LSOPC	47.61 ± 0.30^b^	70.13 ± 0.41^b^	92.83 ± 0.15^b^	15.23 ± 0.20^b^
RS + 6% LSOPC	48.22 ± 0.52^b^	70.62 ± 0.29^b^	93.32 ± 0.41^b^	12.65 ± 0.42^c^
RS + 9% LSOPC	40.45 ± 0.44^c^	63.36 ± 0.48^c^	85.61 ± 0.23^c^	13.12 ± 0.22^c^

*The data are given as mean ± S.D. (n = 3). Different letters indicated a significant difference (p < 0.05)*.

### Effect of LSOPC on the Texture Characteristic of RS

Qiu et al. (22) found the increase of hardness was a typical retrogradation behavior of glutinous rice. As shown in Table 2, the addition of LSOPC decreased the hardness of RS gel, and the decline was positively related to the amount of LSOPC added. It indicated that procyanidins could inhibit RS retrogradation to a certain degree. A possible reason for this was that procyanidins lessened the recrystallization level of amylopectin, and thus reduced the stiffness of the starch granule and delayed the brittle hardening of mechanical properties of the macroscopical system.

**Table 2:** Texture characteristic of RS with or without LSOPC.

**Sample**	**Hardness**	**Springiness**	**Cohesiveness**	**Gumminess**	**Chewiness**
RS	0.75 ± 0.09^a^	1.03 ± 0.16^a^	0.76 ± 0.03^a^	0.43 ± 0.06^a^	0.52 ± 0.02^a^
RS + 3% LSOPC	0.64 ± 0.08^a^	0.85 ± 0.05^ab^	0.68 ± 0.04^b^	0.40 ± 0.05^a^	0.29 ± 0.05^b^
RS + 6% LSOPC	0.21 ± 0.05^b^	0.79 ± 0.08^b^	0.68 ± 0.01^b^	0.14 ± 0.04^b^	0.11 ± 0.02^c^
RS + 9% LSOPC	0.16 ± 0.02^b^	0.70 ± 0.03^b^	0.61 ± 0.02^c^	0.11 ± 0.04^b^	0.08 ± 0.01^c^

*The data are given as mean ± S.D. (n = 3). Different letters indicated a significant difference (p < 0.05)*.

### LF-NMR Properties of RS With or Without LSOPC

**Figure 1:**
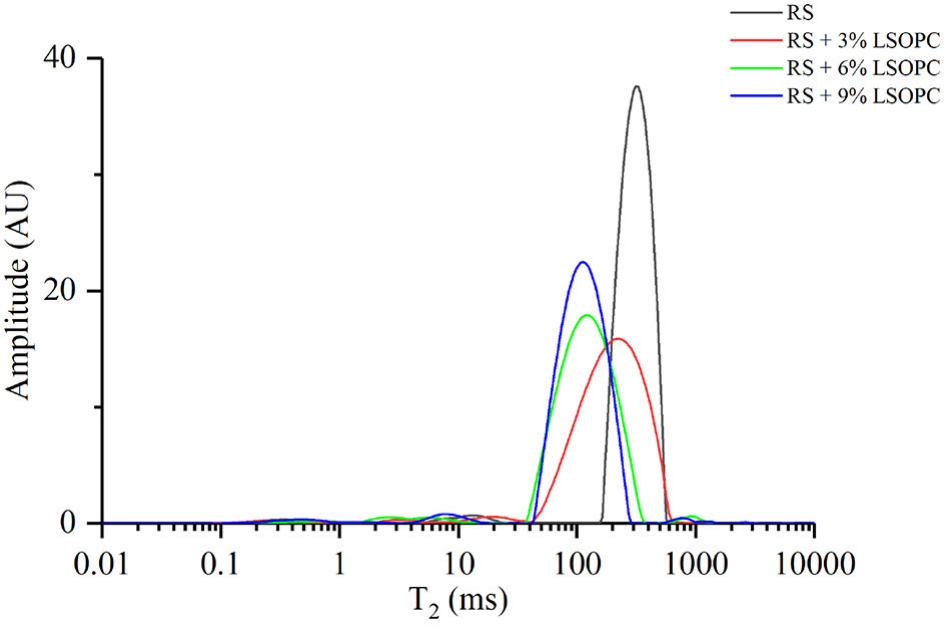
LF-NMR spectra of RS with or without LSOPC.

The changes in water distribution of samples were determined by using LF-NMR analysis, and the corresponding spectra were shown in Figure 1. The length of relaxation time was inversely associated with the degree of tightness of the combination of water and matter (23). A long relaxation time indicated the proton had a higher degree of freedom and was easily discharged. The condition of the proton with a short relaxation time was the opposite. Hence the migration rules of water in RS could be analyzed according to the changes of relaxation time. Generally, the relaxation time could be divided into three periods: T_21_ (0.02–10 ms) corresponded to the binding water, which bound the matter most tightly; T_22_ (17–165 ms) matched the water that did not flow easily, which was between the binding water and the free water; and T_23_ (180–1,400 ms) corresponded to the free water, which possessed the highest degree of fluidity. Figure 1 showed the amplitude of RS without LSOPC was the largest at T_23_, indicating the internal water was mainly free water. With the increase of LSOPC addition, the wave peak shifted to the left and finally located at T_22_, suggesting the water flow became harder. The results of LF-NMR showed procyanidins could increase the water holding capacity of RS, and subsequently hinder its recrystallization.

### FT-IR Properties of RS With or Without LSOPC

**Figure 2:**
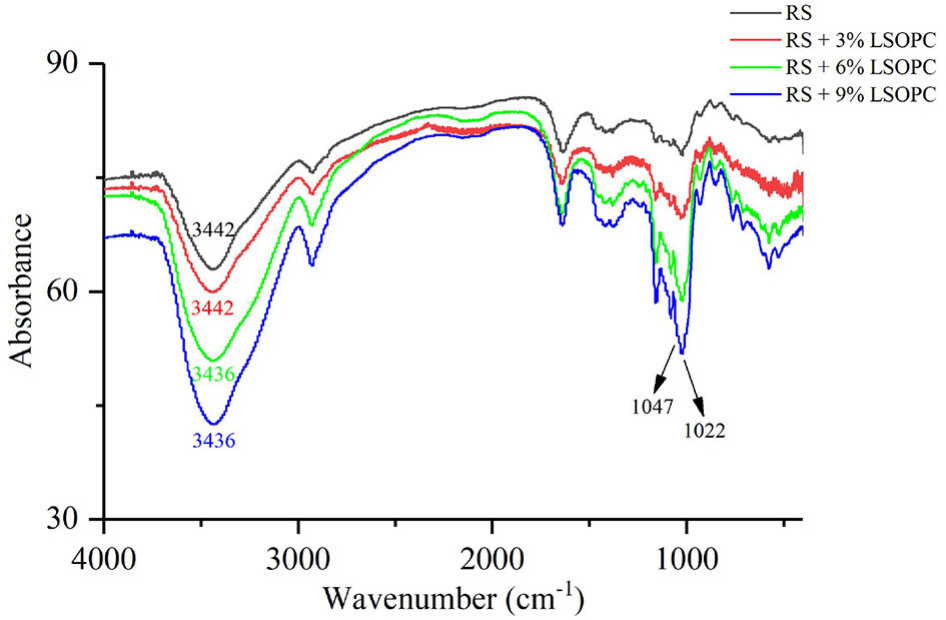
Infrared spectroscopy spectra of RS with or without LSOPC.

The infrared spectra of samples are presented in Figure 2, and there was an absorption peak at 3,442 cm^−1^ for RS. Notably, the absorption peak did not change significantly after adding 3% LSOPC, but moved toward the direction of low wavenumber after adding 6 or 9% LSOPC. The band of 3,100–3,500 cm^−1^ in the infrared spectrum could reflect the hydrogen bond strength formed by intramolecular hydroxyl groups: the stronger the hydrogen bond strength, the larger the shift of the spectral band to the lower wavenumber (24, 25). It demonstrated that a higher proportion of procyanidins could increase the hydrogen bond strength of the system. Retrogradation is a process in which starch molecules rearrange and combine to become orderly after gelatinization. The procyanidins would interact with the starch's molecular chain to strengthen the hydrogen bonds of system and make starch retrogradation more difficult. In addition, the band of 1,047 cm^−1^ correlated with the orderly area of the starch, while the band of 1,022 cm^−1^ related to the amorphous area of the starch. The ratios of absorbance values from the infrared spectra at 1,047 and 1,022 cm^−1^ could reflect the orderly degree of the crystal region (26). As shown in Table 3, the ratio decreased after adding 6 or 9% LSOPC. These results indicated a higher proportion of procyanidins performed better at inhibiting the retrogradation of RS.

**Table 3:** Ratios of absorbance values from the infrared spectra at 1,047 and 1,022 cm^−1^.

**Sample**	**Ratios**
RS	0.915
RS + 3% LSOPC	0.908
RS + 6% LSOPC	0.852
RS + 9% LSOPC	0.843

### XRD Properties of RS With or Without LSOPC

**Figure 3:**
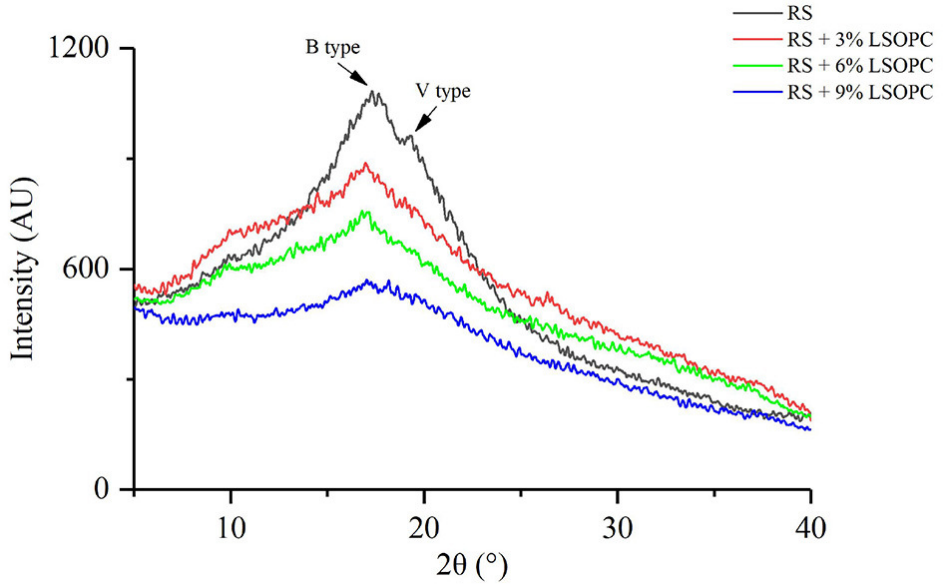
X-ray diffraction patterns of RS with or without LSOPC.

The crystal properties of samples were measured by using XRD analysis (Figure 3). The diffraction peaks of RS appeared at 17° and 20°. The former represented the B type crystallization of starch, and the latter reflected the V type crystallization of starch. The generation of a V type crystal might be due to the formation of the complex between amylose and lipid (27). Nevertheless, the diffraction peak intensity of RS at 17° decreased after the addition of LSOPC, and the decreasing range rose with an increase of the LSOPC addition. The hydroxyl group, which was introduced by procyanidins, could have interacted with the molecular chain of the starch through hydrogen bonds (28). Therefore, procyanidins hampered the movement of water molecules near starch, lessened the number of water molecules that effectively participated in the retrogradation, and reduced the crystallinity of the starch.

### SEM Properties of RS With or Without LSOPC

**Figure 4:**
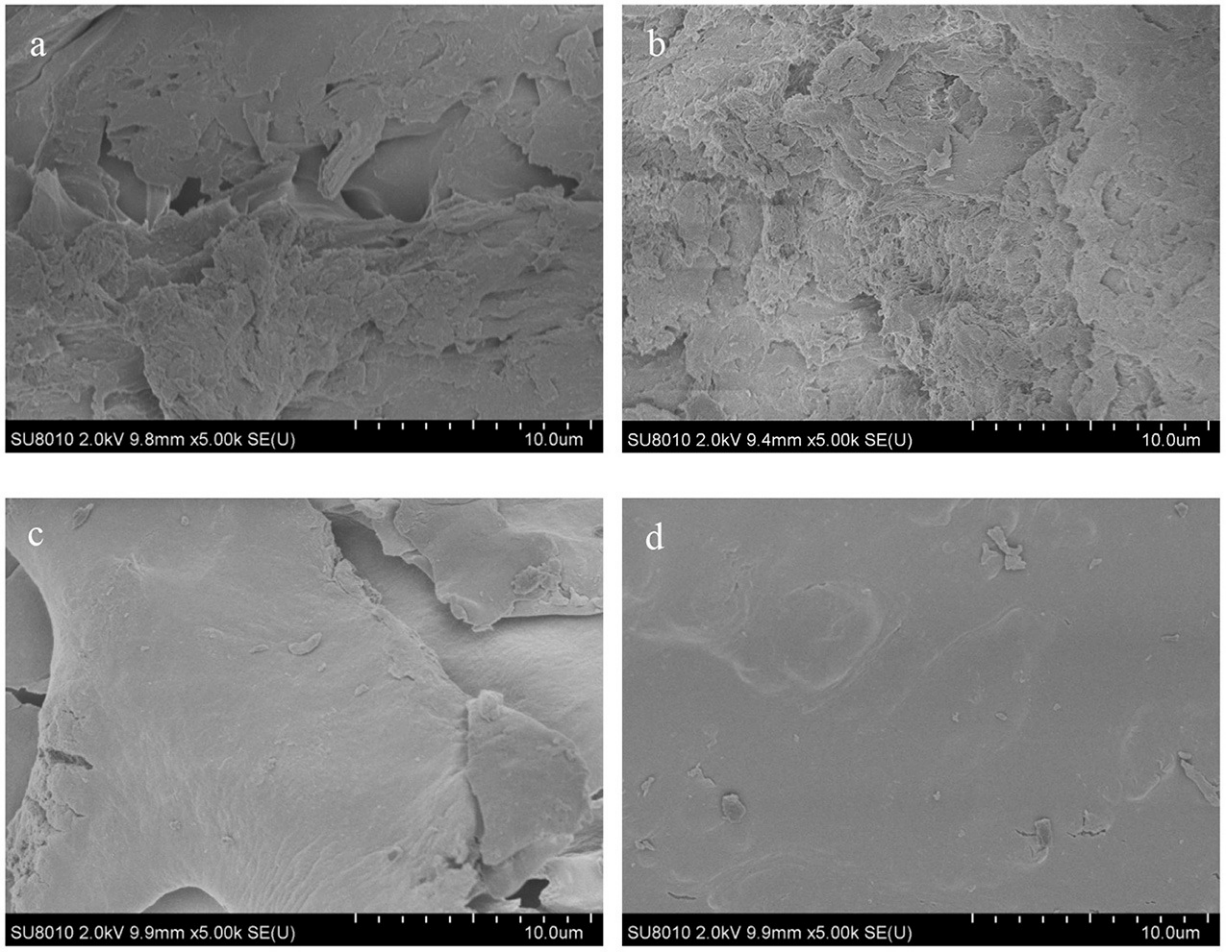
Scanning electron micrographs of RS with or without LSOPC, RS **(A)**, RS + 3% LSOPC **(B)**, RS + 6% LSOPC **(C)**, and RS + 9% LSOPC **(D)**.

The scanning images of RS with or without LSOPC are displayed in Figure 4. There were many starch fragments with different shapes piled together, and no obvious change on starch surface occurred after adding 3% LSOPC. However, starch fragments were reduced and became smaller with the addition of 6% LSOPC. It indicated procyanidins reduced the proportion of starch fragments and enabled the whole to be flatter and more compact. Moreover, when 9% LSOPC was added, the starch fragments were further reduced and the surface smoothness was further enhanced. The probable reason was procyanidins competed with starch granules for the nearby water molecules, resulting in the low degree of pasting completion of starch granules (29). Thereby some starch granules had compact structures, which was not beneficial to ordering of starch molecules and hindered the retrogradation.

### Effect of LSOPC on the Dynamic Rheological Properties of RS

**Figure 5:**
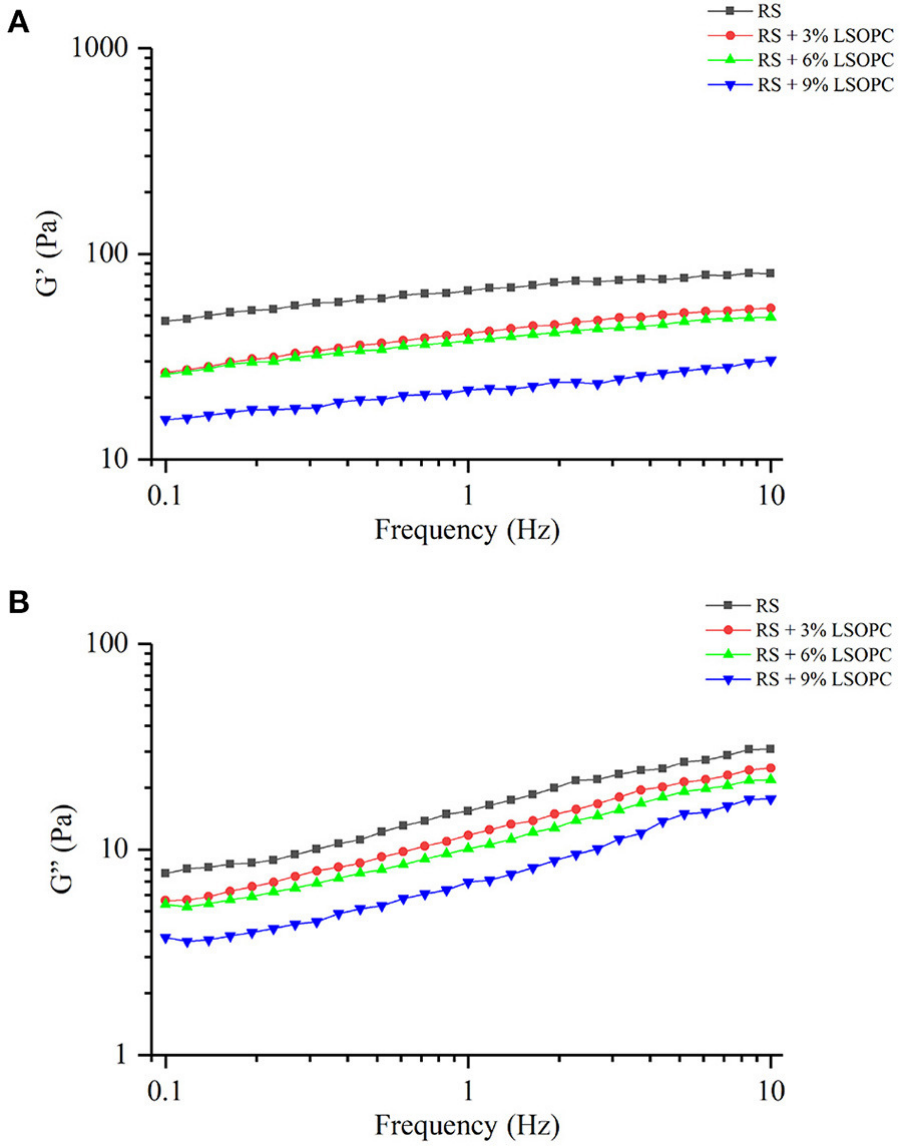
Dynamic rheological properties of RS with or without LSOPC, G' **(A)**, G” **(B)**.

As presented in Figure 5, the effects of LSOPC on the dynamic rheological properties of RS were determined. G' was the storage modulus, representing the energy stored in the matter during deformation. It could reflect the ability to restore to the original state, and the storage modulus was positively correlated to the restoring ability. It was reported that G' was linked to the amylose aggregation in the early stage of retrogradation (30). G” was the loss modulus, representing the energy consumed by the matter during deformation due to the resistance of viscous drag. It could mirror the capacity to resist flow, and the loss modulus positively correlated to the resisting capacity. Figure 5 showed G' was larger than G” in all groups, and both of them tended to rise with the increase of frequency. Compared with RS, the RS-LSOPC groups revealed lower G' and G”, demonstrating the dynamic moduli declined by the addition of procyanidins. A possible reason may be that procyanidins contended with the starch for the nearby water molecules, decreasing the amylose exudation and forming a weak gel structure (31). Overall, procyanidins had an anti-retrogradation effect on starch in the early stage.

### Simulation Study of Molecule

**Figure 6:**
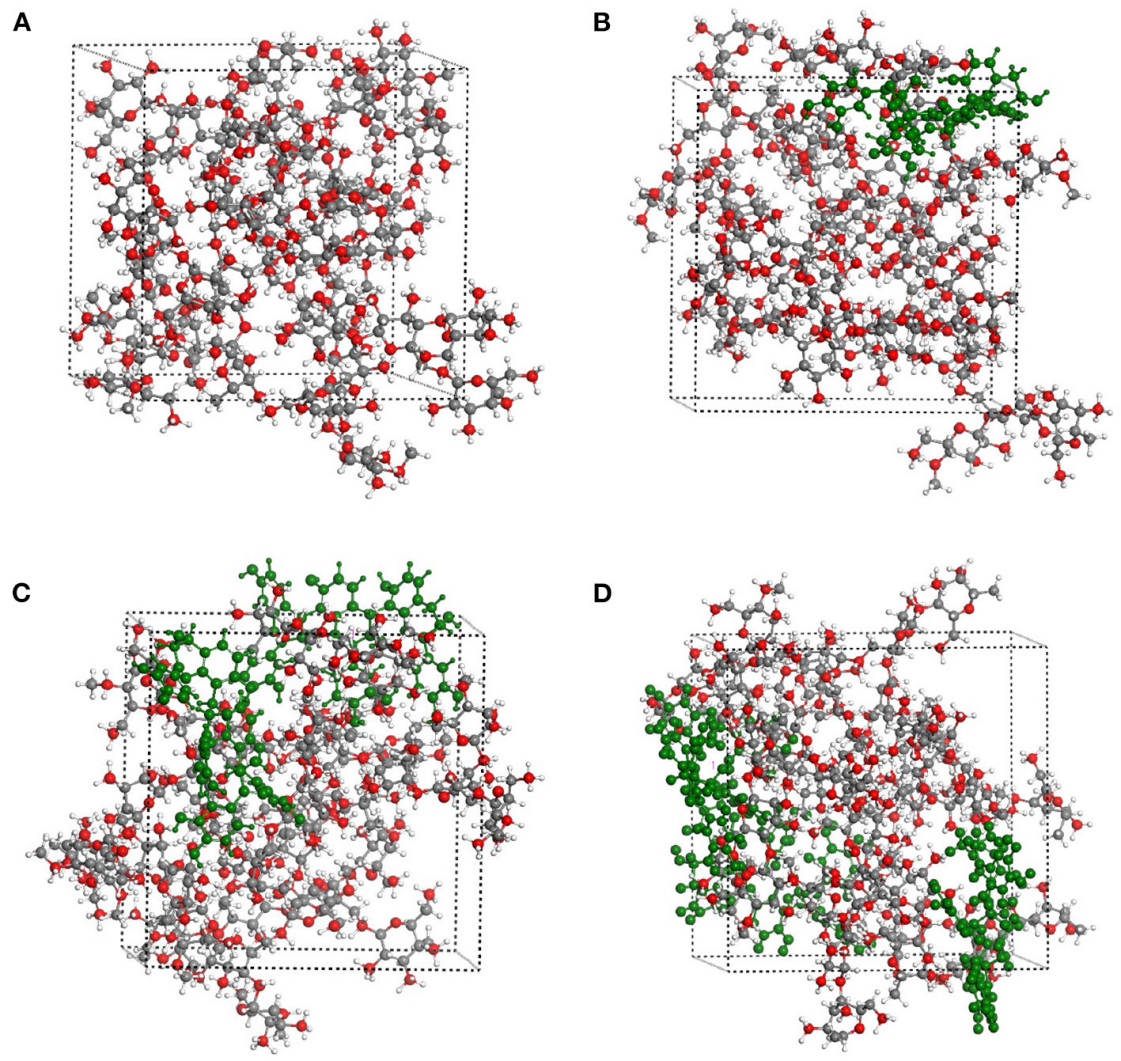
Simulation systems of molecule of RS with or without LSOPC, 0PC S **(A)**, 1PC S **(B)**, 2PC S **(C)**, and 3PC S **(D)**.

**Figure 7:**
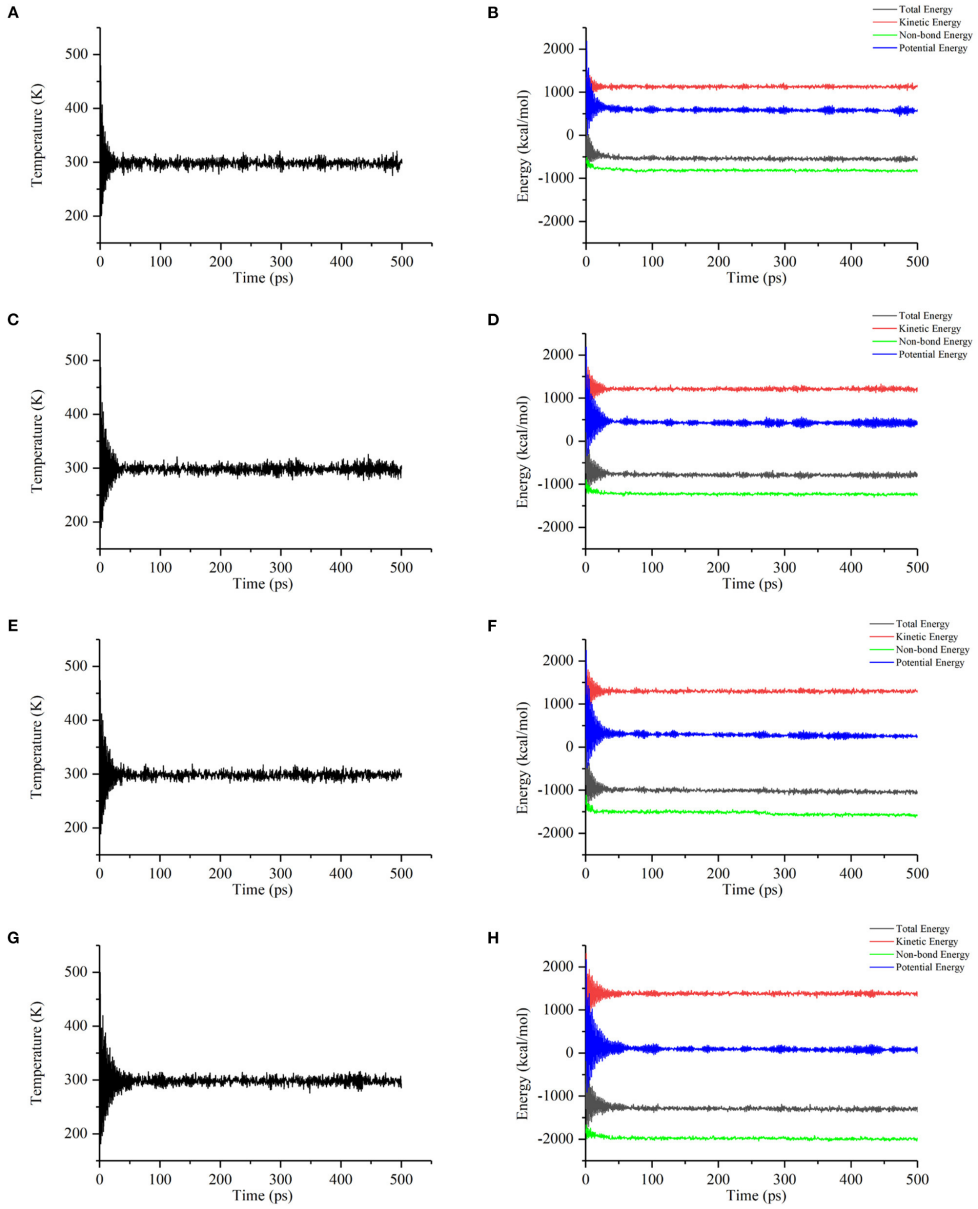
Changes of temperature and energy with time in simulation systems, temperature change of 0PC S **(A)**, energy change of 0PC S **(B)**, temperature change of 1PC S **(C)**, energy change of 1PC S **(D)**, temperature change of 2PC S **(E)**, energy change of 2PC S **(F)**, temperature change of 3PC S **(G)**, and energy change of 3PC S **(H)**.

**Figure 8:**
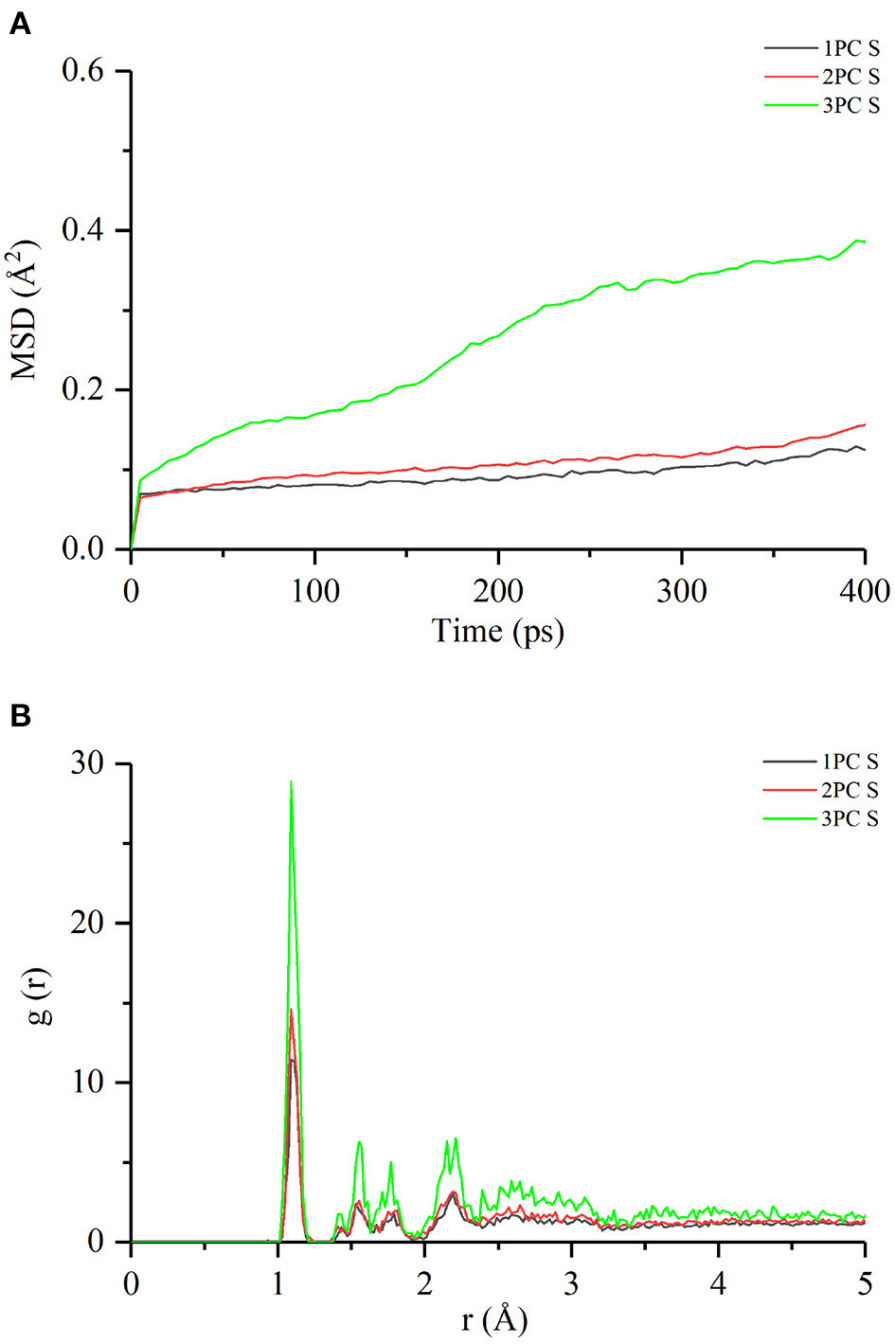
Mean square displacement **(A)** and radial distribution function **(B)** of RS with or without LSOPC.

**Figure 9:**
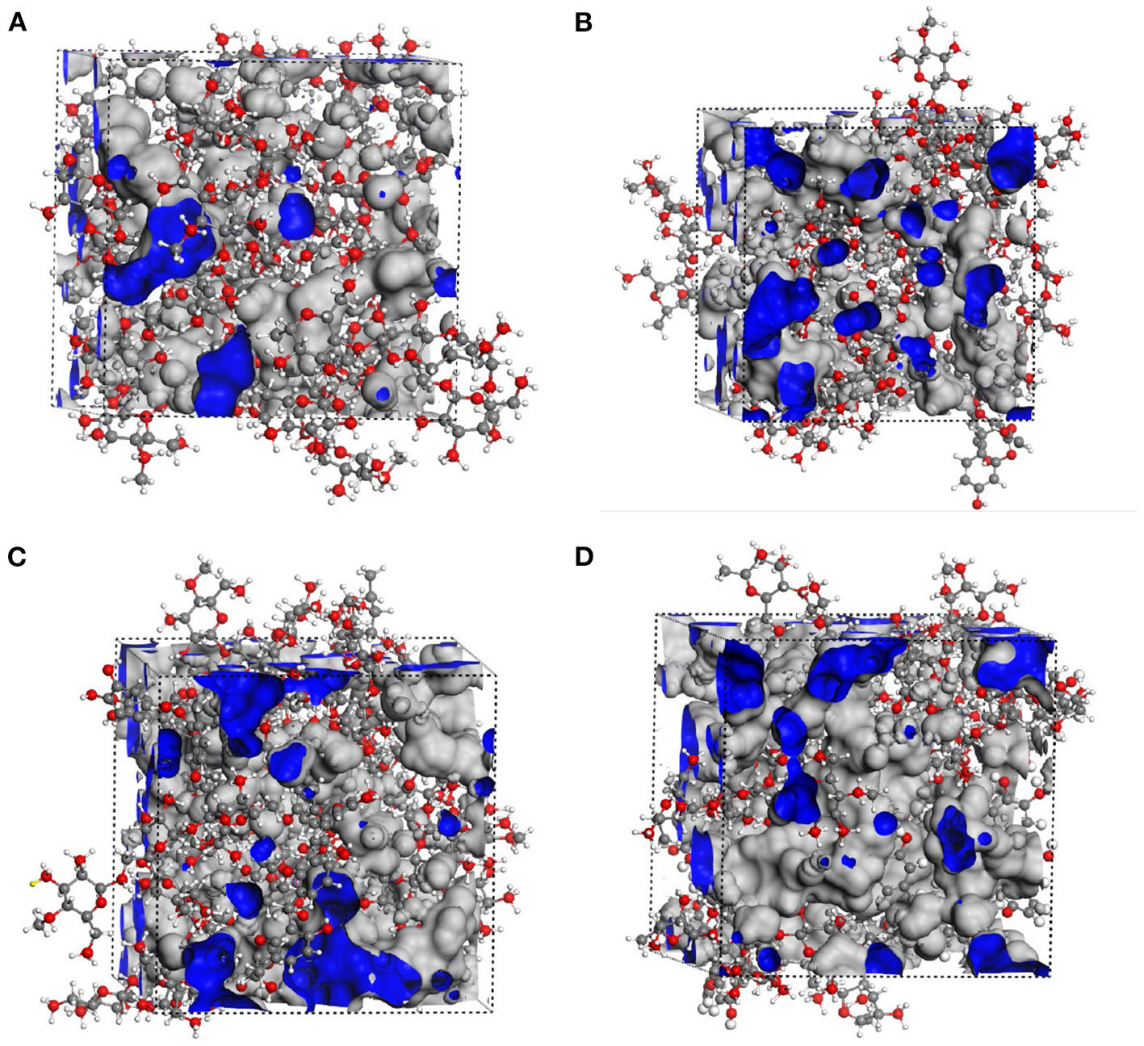
Free volume distribution of RS with or without LSOPC, 0PC S **(A)**, 1PC S **(B)**, 2PC S **(C)**, and 3PC S **(D)**.

The interactions between starch and procyanidins were simulated using Materials Studio 8.0. Molecular interaction volume models were shown in Figure 6. The content of procyanidins was 0% (0PC S), 3% (1PC S), 6% (2PC S), and 9% (3PC S), respectively. As displayed in Figure 7, the temperature fluctuation of the four systems was not obvious after a long dynamic simulation. Besides, with the increase of the procyanidins ratio, the potential energy, non-bond energy, and total energy of the system decreased, while the kinetic energy increased slightly. It indicated that the addition of procyanidins enhanced the stability of system, but whether the movement ability was improved still needs further verification. Mean square displacement refers to the degree that the spatial position of molecules in the simulation system deviates from the initial position at a certain time, which is used to characterize the movement ability of a molecular chain (32, 33). As exhibited in Figure 8A, the mean square displacement of the system increased over time, and the increment was proportional to the ratio of procyanidins. It showed the addition of procyanidins promoted the movement ability of the starch chain. Radial distribution function is employed to describe the molecular dynamics of the system. The probability of finding a particle at a certain distance around another particle can be obtained by this function. The radial distribution function is helpful in describing the structure of the molecular system and can characterize the molecular interactions of a simulation system (34). The peak intensity comparison of 1.1 Å was 3PC S > 2PC S > 1PC S (Figure 8B), which implied the higher the content of procyanidins, the stronger the interaction force. It was consistent with the results of FT-IR analysis. According to the theory of free volume, the volume of a solid or liquid includes occupied volume and free volume. The former is the volume occupied by molecules themselves, and the latter is the space between molecules. Free volume is distributed in the polymer irregularly, which provides the active space for molecules. Notably, the free volume can significantly affect the movement ability of a polymer (35). The free volume distribution of 0PC S, 1PC S, 2PC S, and 3PC S are presented in Figure 9, the percentage was 26.71, 35.77, 39.97, and 41.78%, respectively. It means the movement ability of the starch chain improved as the procyanidins ratio increase, which confirmed the results of mean square displacement.

## Conclusion

This study researched the effects of LSOPC on the retrogradation of RS. The results of texture characteristics showed LSOPC could reduce the degree of retrogradation of starch. DSC analysis indicated LSOPC were able to decrease the energy required for starch gelatinization. The reordering and recrystallization processes of starch molecules were effectively hampered by LSOPC, which was demonstrated by FT-IR, SEM, LF-NMR, and XRD analyses. The formation of a weak gel structure between LSOPC and RS was illustrated by the analysis of the results of the dynamic rheological properties. Molecule simulation implied LSOPC could enhance the stability of system and the movement ability of the starch chain. In short, the retrogradation of RS could be obviously retarded, and the effects were positively correlated with the procyanidins ratio. The study shows that procyanidins could be useful for the anti-retrogradation of rice products.

## Data Availability Statement

The original contributions presented in the study are included in the article/supplementary material, further inquiries can be directed to the corresponding author/s.

## Author Contributions

NF: conceptualization, methodology, data curation, and writing-original draft. SS: data curation and methodology. HH: investigation and funding acquisition. ST: investigation and visualization. JT: data curation. QW: conceptualization, writing-review and editing, supervision, and funding acquisition. JX: supervision and funding acquisition. All authors contributed to the article and approved the submitted version.

## Funding

This work was financially supported by National Natural Science Foundation of China (Nos. 32001705 and 21908048), State Key Laboratory of Marine Resource Utilization in South China Sea (Hainan University) (No. MRUKF2021002), Key Laboratory of Food Nutrition and Functional Food of Hainan Province (No. KF202009), and the Collaborative Grant-in-Aid of the HBUT National 111 Center for Cellular Regulation and Molecular Pharmaceutics (No. XBTK-2020005).

## Conflict of Interest

The authors declare that the research was conducted in the absence of any commercial or financial relationships that could be construed as a potential conflict of interest.

## Publisher's Note

All claims expressed in this article are solely those of the authors and do not necessarily represent those of their affiliated organizations, or those of the publisher, the editors and the reviewers. Any product that may be evaluated in this article, or claim that may be made by its manufacturer, is not guaranteed or endorsed by the publisher.
